# Cfa-circ002203 was upregulated in rapidly paced atria of dogs and involved in the mechanisms of atrial fibrosis

**DOI:** 10.3389/fcvm.2023.1110707

**Published:** 2023-08-01

**Authors:** Wenfeng Shangguan, Tianshu Gu, Rukun Cheng, Xing Liu, Yu Liu, Shuai Miao, Weiding Wang, Fang Song, Hualing Wang, Tong Liu, Xue Liang

**Affiliations:** ^1^ Tianjin Key Laboratory of Ionic-Molecular Function of Cardiovascular Disease, Department of Cardiology, Tianjin Institute of Cardiology, The Second Hospital of Tianjin Medical University, Tianjin, China; ^2^ Department of Cardiology, Taikang Ningbo Hospital, Ningbo, China; ^3^ Department of Geriatric, The Second Hospital of Tianjin Medical University, Tianjin, China

**Keywords:** atrial fibrillation, atrial fibrosis, high-throughput sequencing, bioinformatics, cfa-circ002203

## Abstract

**Background and aims:**

The role of circular RNAs (circRNAs) in the pathophysiology of cardiovascular disease is gradually being elucidated; however, their roles in atrial fibrillation (AF)-related fibrosis are largely unknown. This study aimed to characterize the different circRNA profiles in the rapid-pacing atria of dogs and explore the mechanisms involved in atrial fibrosis.

**Methods:**

A rapid right atrial-pacing model was established using electrical stimulation from a pacemaker. After 14 days, atrial tissue was collected for circRNA sequencing analysis. *In vitro* fibrosis was established by stimulating canine atrial fibroblasts with angiotensin II (Ang II). The fibroblasts were transfected with siRNA and overexpressing plasmids to explore the effects of cfa-circ002203 on fibroblast proliferation, migration, differentiation, and the expression of fibrosis-related proteins.

**Results:**

In total, 146 differentially expressed circRNAs were screened, of which 106 were upregulated and 40 were downregulated. qRT-PCR analysis showed that cfa-circ002203 was upregulated in both *in vivo* and *in vitro* fibroblast fibrosis models. The upregulation of cfa-circ002203 enhanced proliferation and migration while weakening the apoptosis of fibroblasts. Western blotting showed that cfa-circ002203 overexpression increased the protein expression levels of fibrosis-related indicators (Col I, Col III, MMP2, MMP9, and α-SMA) and decreased the protein expression levels of pro-apoptotic factors (Bax and Caspase 3) in Ang II-induced fibroblast fibrosis.

**Conclusion:**

Cfa-circ002203 might serve as an active promoter of the proliferation, migration, and fibrosis of atrial fibroblasts and is involved in AF-induced fibroblast fibrosis.

## 1. Introduction

Atrial fibrillation (AF) is the most common arrhythmia and is associated with heart failure, which leads to high morbidity and mortality (1). The long-term persistence of AF may result in the remodeling and deposition of atrial fibrous tissue. Cardiac fibrosis is one of the major factors resulting in cardiac remodeling in patients with AF (2, 3). In addition, endomysial fibrosis is the strongest determinant of AF complexity compared with other structural alterations (4). Cardiac fibrosis is characterized by notable changes in the synthesis and degradation of collagen I and III, the main components of the extracellular matrix (ECM), and the malfunction of matrix metalloproteinases (MMP2 and MMP9) (5). The cellular and molecular mechanisms underlying the development of cardiac fibrosis have not been completely elucidated. Studies have indicated that phenotypic changes in cardiac fibroblasts, a class of small fusiform cells that control the composition and structure of the ECM, contribute to AF-induced cardiac fibrosis. When activated by pro-fibrotic stimulation, such as mechanical stress, and growth factors, such as angiotensin II (Ang II), fibroblasts proliferate and differentiate into a secretory phenotype, namely myofibroblasts, with α-smooth muscle actin (α-SMA) expression acting as a marker of the myofibroblast phenotype (6). Consequently, cardiac fibrosis might directly involve in the occurrence and perpetuation of AF and its related disease.

Cellular circular RNAs (circRNAs) are a class of stable, single-stranded RNAs with covalently closed head-to-tail circularized transcripts (7). Owing to improvements in RNA sequencing and bioinformatics tools, thousands of circRNAs in various organisms have been identified. CircRNAs play an important role in the pathogenesis of heart disease, as previous studies have revealed that they play regulatory roles in heart failure (8, 9) and pathological hypertrophy (10). For example, Ni et al. found that circHIPK3 inhibited Ang II-induced cardiac fibrosis by sponging miR-29b-3p (11). In a diabetic db/db mouse model, circRNA_010567 was found upregulated and knockdown of circRNA_010567 could reduce synthesis of Col I, Col II and α-SMA in Ang II-treated cardiac fibroblasts (12). Wu et al. uncovered that circYAP was significantly decreased in the hearts of patients with cardiac hypertrophy and the pressure overload mouse model (13). Most of these studies based on cells or mice. Our research group previously performed circRNA sequencing analysis in the atrial tissue of a rapid atrial pacing dog model and screened several differentially expressed circRNAs (14), indicating that they may be involved in the pathology of AF and fibrosis. Based on this, this study focuses on the function of the molecular mechanism of circRNA's involvement in the fibrosis of atrial fibroblasts. Given the extensive influence of circRNAs on the activity of microRNA (miRNA), there is great interest in understanding the effect of circRNAs on the gene regulatory functions of miRNAs (15). In this molecular mechanism, circRNAs act as sponges and compete with miRNAs to bind to the untranslated regions of messenger RNA (mRNA) and regulate the expression of target genes. As post-transcriptional regulators, circRNAs participate in mammalian physiological and pathological processes. Each circRNA acts as a competing endogenous RNA (ceRNA) with multiple miRNAs, generating a complex and fine-tuned system that regulates the pathology of diseases.

Based on the above information, the objective of this study was to identify circRNAs associated with AF in dogs by producing an interaction network diagram of circRNA-miRNA-mRNA and performing qRT-PCR verification, and to further identify the role of circRNAs in fibroblast inflammation, proliferation, and fibrosis in an *in vitro* atrial fibroblast fibrosis model induced by Ang II.

## 2. Methods

### 2.1. Rapid right atrial pacing model establishment

This study was conducted in compliance with the ARRIVE guidelines and approved by the Laboratory Animal Ethics Committee of the Institute of Radiation Medicine, Chinese Academy of Medical Sciences (approval number: IRM-DWLL-2019018).

Twelve healthy mongrel dogs of either sex weighing between 12 and 14 kg were randomly divided into two groups, a control group (*n* = 6) and an AF group (*n* = 6) with the established rapid right atrial pacing model. After randomization, sex and body weight were equally distributed between the two groups by artificial adjustment to reduce system errors. The dogs were fasted for 12 h and anesthetized with an intravenous injection of 3% (w/v) isopentobarbital sodium (dissolved in normal saline, 30 mg/kg) into the right upper limb. After endotracheal intubation, an ALC-V8 animal ventilator (tidal volume 12–15 ml/kg, frequency 25 bpm; Shanghai Alcott Biotech Co., Ltd, Shanghai, China) was connected to maintain mechanical ventilation. The dog was fixed in the supine position on the operating table, and their electrocardiography (ECG), blood pressure, and oxyhemoglobin saturation were monitored using a multichannel electrophysiological system (Shanghai Hongtong Industrial Co. Ltd., Shanghai, China) during anesthesia before and after 14 days of pacing. A longitudinal incision of 3 cm was made on the right side of the middle of the trachea, a modified pacemaker electrode (St. Jude Medical, St. Paul, MN, USA) was inserted along the external jugular vein into the right atrium, and electrical stimulation of 5 V and 200 bpm was applied using an electrophysiological stimulator (Suzhou Oriental Electronic Instrument Factory, Suzhou, China). The pacing frequency was adjusted to 500 bpm, and the electrode was fixed when the pacing was good. A surgical incision was made in the interscapular region and a pacemaker pocket was buried subcutaneously. Electrical stimulation with a pacing voltage of 5 V, pulse width of 0.2 ms, and frequency of 500 bpm was applied continuously. The control group underwent the same operation as described above, with pacemaker placement, but no electrical stimulation. Aseptic procedures were strictly followed during the operation, and antibiotics (1 g of cefuroxime sodium dissolved in 50 ml of normal saline) were administered to each dog by an intravenous drip three times a day for 3 days after the operation to prevent pacemaker pocket infection.

After 14 days of pacing, the anesthetized dog was fixed on the operating table, the pacing electrode was removed, and the mapping electrodes (diameter:1.5 mm; distance between poles:1.5 mm) were stitched onto the left and right atrial epicardia and four limbs to monitor the atrial epicardial electrocardiography and limb-lead electrocardiography using a multi-channel electrophysiological recorder. AF susceptibility was assessed according to preprocedural cardiac stimulation (S1S2). AF was induced by four times the threshold and 600 bpm rapid stimulation for 2 min, and was regarded as successful when the electrocardiogram sinus *P* wave was replaced by a rapid and disorderly fibrillation wave and the duration of this electrical activity disorder was more than 1 s.

### 2.2. Tissue preparation for sequencing

The dogs were euthanized by an intravenous injection of overloaded 3% isopentobarbital sodium (85 mg/kg). The hearts of the experimental dog in each group were quickly removed and washed with pre-cooled phosphate-buffered saline (PBS) at 4°C. The right atrium was separated and weighed. The right atria of three dogs from each group were randomly fixed in 10% neutral formalin for histomorphological examination. The remaining tissue of the right atrium was placed in a cryopreservation tube and stored at −80°C for qRT-PCR detection. The remaining three atrial tissues of dogs were washed with pre-cooled PBS solution at 4°C and then placed in a cryopreservation tube and stored in the refrigerator at −80°C until the total RNA was extracted for high-throughput sequencing.

### 2.3. Hematoxylin-Eosin (HE) staining and Masson staining

HE staining and Masson staining were performed using a HE staining kit (Solarbio, Beijing, China) and Masson staining kit (Solarbio, Beijing, China), respectively, on the right atria fixed in 10% neutral formalin. Briefly, the atria were dehydrated using an ethanol gradient, cleared with xylene, and embedded in paraffin. The paraffin-embedded atria were cut into 5-µm sections and dewaxed with xylene, hydrated with an ethanol gradient, and stained with hematoxylin and eosin or Masson trichrome. The sections were observed under an optical microscope (Olympus, Tokyo, Japan) at a magnification of 200×.

### 2.4. High-throughput sequencing

The total RNA was isolated using Trizol Reagent (Invitrogen, Carlsbad, CA, USA) and resuspended in sterile water for RNA quality control. The RNA purity is suitable for sequencing when the OD260/OD280 value ranges from 1.8 to 2.1. Ribosomal RNAs (rRNAs) were removed using RiboZero rRNA removal kits (Illumina, San Diego, CA, USA). A circRNA sequencing library was constructed using the TruSeq Stranded Total RNA Library Prep Kit (Illumina, San Diego, CA, USA). Library quality control was performed using a BioAnalyzer 2,100 instrument. CircRNA sequencing was performed in the two-terminal mode on an Illumina HiSeq 4,000 sequencer (Illumina, San Diego, CA, USA). Q30 was the quality control standard, and Q30 > 80% indicates good sequencing quality. Cutadapt software (version 1.9.3) was used to remove connectors and obtain high-quality reads for statistical analysis.

For circRNA expression profile screening, clean reads were compared with the dog reference genome (UCSC canFam3) using Bowtie2 software, and, under the guidance of Ensembl Transcriptome GTF files, find_circ software (7) was used for circRNA detection. The number of spliced reads reflects the abundance of circRNAs. The identified circRNAs were annotated using the circBase database (16) according to their genomic locations. In this study, the standardized reads number was used to screen differentially expressed circRNAs between two groups with multiple changes of ≥2.0 and *P* ≤ 0.05 as the threshold of differential circRNAs. The differentially expressed circRNAs screened from the atrial tissue were analyzed by clustering with fragments per kilobase of exon model per million mapped fragment values using the heatmap2 function of R. A circRNA-miRNA-mRNA network diagram was constructed using Cytoscape software based on the binding information available in online databases, including TargetScan, miRNet, and ENCORI (Starbase V3.0), and the relationship between them was visualized.

### 2.5. Culture of atrial fibroblasts

The myocardial tissue from the posterior wall of the right atrium was cut into 1 × 1 × 1-cm pieces. After the residual blood was washed with PBS, the pieces were placed in Petri dishes in RPMI-1640 medium. The tissue was digested with 0.125% trypsin, prepared into a suspension, and cultured in a culture flask at 37°C. After 1.5 h of culturing, the medium was replaced with fresh medium every other day. Cells were passaged at the logarithmic growth stage until they reached 90% confluence. The mixture in the culture flask was digested with 0.25% trypsin. When the cells became spherical, PBS was added to stop the digestion. The cell suspension was centrifuged at 1,500 rpm for 5 min, and the cell precipitate was suspended. The cells were passaged at a ratio of 1:2 and cultured in a cell incubator. Two to three generations of fibroblasts were used in subsequent experiments.

### 2.6. *In vitro* Ang-II induced fibroblast fibrosis

The fibroblasts were maintained in RPMI-1640 (supplemented with 10% fetal bovine serum, 2 mm L-glutamine, 100 U/ml penicillin G, and 100 µg/ml streptomycin) (Invitrogen, Carlsbad, CA, USA) and cultured at 37°C in a humidified incubator with 5% CO_2_ (Thermo Fisher, Waltham, MA, USA). Fibroblasts were seeded in a six-well plate at 5 × 10^4^ cells/well. After 12 h of culture, the medium was replaced with the L-15 basic medium without serum and penicillin-streptomycin (Gibco, Carlsbad, CA, USA). After 12 h of culture, 5.0 µmol/L angiotensin II (AngII) (ab120183, Abcam, Waltham, MA, USA) was added to stimulate the fibroblasts. The cells were collected at various time points for subsequent detection.

### 2.7. Cell transfection

The cells were seeded in six-well plates at a density of 2 × 10^6^ cells/well, and basic culture medium without penicillin–streptomycin was added to each well. When the cells grew to about 80% confluence, 200 pmol of siRNA or 4 µg of pcDNA3.1+ plasmid (Invitrogen, Carlsbad, CA, USA) plus 10 µl of lipofectamine™2000 (Invitrogen, Carlsbad, CA, USA) were added to each well. The six-well plate was placed in a cell incubator at 37°C for 6–8 h and then replaced with complete medium. Twenty-four hours after transfection, Ang II was added and the cells were collected after 24 h for subsequent tests.

### 2.8. Western blot

To each well of six-well plates, 200 µl of prepared cell lysate was added (radio immunoprecipitation assay: phenylmethanesulfonyl fluoride = 100:1) and the plates were shaken slowly at 4°C for 30 min. Protein samples were obtained by centrifugation, quantified using a bicinchoninic acid kit (Sigma-Aldrich, St. Louis, MO, USA), and loaded into sodium dodecyl sulfate polyacrylamide gel electrophoresis mixed with loading buffer. Proteins were transferred from the gels to the polyvinylidene fluoride (PVDF) membranes using a Bio-Rad Trans-Blot Turbo system (Hercules, CA, USA). After transfer, the PVDF membrane was placed in 5% defatted milk powder dissolved in TBST-Tween-20, blocked at room temperature for 1 h, and incubated overnight with primary antibodies. On the second day, the rinsed PVDF membranes were immersed in the secondary antibodies and incubated at room temperature for 1 h. Enhanced chemiluminescence was added to the PVDF membrane and developed and photographed using a Gel Imaging System. Detailed information on the primary and secondary antibodies is provided in Table 1.

**Table 1:** Primary and secondary antibodies used in western blot.

Primary antibodies	Dilution	Solvent
Rabbit Anti-Collagen I antibody (ab233080)	1:1,000	TBST
Rabbit Anti-Collagen III antibody (ab7778)	1:5,000	TBST
Rabbit anti-MMP2 (ab97779)	1:2,000	TBST
Rabbit anti-MMP9 (ab219372)	1:1,000	TBST
Goat anti-α-SMA (ab21027)	1:1,000	TBST
Rabbit anti-TIMP1 (ab216432)	1:1,000	TBST
Rabbit anti-POSTN (ab92460)	1:1,000	TBST
Rabbit anti-Bax (ab104156)	1:1,000	TBST
Mouse anti-Bcl2 (ab692)	1:1,000	TBST
Rabbit anti-Caspase3 (ab13874)	1:500	TBST
Mouse anti-β-actin (ab8226)	1:5,000	TBST
Secondary antibodies	Dilution	Solvent
Goat anti-rabbit IgG-HRP (ab7090)	1:5,000	TBST
Goat anti-mouse IgG-HRP (ab97040)	1:5,000	TBST
Donkey anti-goat IgG-HRP (ab7125)	1:5,000	TBST

### 2.9. qRT-PCR

To each well of six-well plates, 1 ml of Trizol reagent (Invitrogen, Carlsbad, CA, USA) was added and lysed for 30 min. Extracted RNA samples were dissolved in sterile water. RNA purity was detected using a NanoDrop ND-2000 instrument, and the OD260/OD280 value ranged from 1.8 to 2.1, indicating that the purity of the RNA was sufficient. RNA was reverse-transcribed into cDNA using a FastQuant RT Kit (with gDNase) (KR106, Tiangen, Beijing, China). The cDNA product served as a template for the PCR using the SYBR Premix Ex Taq II kit (Takara, Kyoto, Japan) on an Applied Biosystems™ 7,500 Fast Dx Real-Time PCR (Thermo Fisher, Waltham, MA, USA). The primer sequences are listed in Table 2.

**Table 2:** Primers used in real-time PCR.

Primer	Species	Sequence (5′-3′)	Tm (°C)
GFI	Dog	F: CGTCATTAACATCGGCATTG	58.0
		R: TGGTCTCCTGGGTGGTAAAG	
cfa-circ001021	Dog	F: GATTATTAGGACACAACGGAGC	58.0
		R: CTGGCAATAATGACTGGTTTCT	
cfa-circ002168	Dog	F: ACCAACTCAGAGTGGGTAA	58.0
		R: GGTCTGAATGATCTGTGGTG	
cfa-circ002203	Dog	F: ATCACATGAACGTTGTCCG	58.0
		R: TGATGGCAACAGCCCTAA	
cfa-circ009305	Dog	F: AACCAAGTACCAAGTGAAGAC	58.0
		R: CAATCATCTGTTCAGGAGTAGT	
Collagen I	Dog	F: TTCAGCTTTGTGGACCTCCG	60.0
		R: GGGTTTCCATACGTCTCGGT	
Collagen III	Dog	F: GTATGAAAGGACATAGAGGCTTTGA	59.0
		R: ACGAGCACCATCGTTACCTC	
MMP2	Dog	F: GTGCTCCACCACCTACAACT	60.0
		R: TGGAAGCGGAACGGGAACT	
MMP9	Dog	F: TCGACGTGAAGACGCAGAC	60.0
		R: TCACACGCCAGTAGAAGCG	
α-SMA	Dog	F: GAATGCTACCACAGCCCTGA	59.0
		R: CCACAACGCAGGTTTCTCTC	
TIMP1	Dog	ACTTGCACAGGTCCCAGA	58.0
		GGGATGGATGAACAGGTAAACA	
POSTN	Dog	TCTCTACTCTTGCTGGTTGTTGT	58.0
		TTTCCTTCCACAGATGGCAC	
Bax	Dog	GTGAGGTCTTCTTCCGAGTGG	60.0
		TCCAGTGTCCAGCCCATGA	
Bcl2	Dog	TCATGTGTGTGGAGAGCGTC	60.0
		TCAAACAGAGGCTGCATGGT	
Caspase3	Dog	CCTGCCGAGGTACAGAACT	59.0
		GCGTATAGTTTCAGCATCGCAC	

### 2.10. Cell scratch test

One day before transfection, cells were seeded into six-well plates at a density of 2 × 10^6^ cells/well and transfected with siRNA or an overexpressed plasmid when the cell density reached approximately 80% influence. After culturing for another 24 h, the cells were scratched in a straight line at an angle perpendicular to the bottom of the culture plate. PBS preheated at 37°C was used to infiltrate the plate and remove the cell debris floating on the cell surface, and the cells were then placed under a microscope to record the size of the scratch space by photography. The basic medium was replaced with Ang II medium. The cells were cultured at 37°C for 24 h. The migration of cells in each well was photographed. At least three images were taken for each group of cells, and “Image-Pro Plus 6.0” software was used to calculate the scratch area and perform statistical analysis.

### 2.11. Immunofluorescence staining

The cells were seeded in glass dishes and transfected with siRNA or an overexpressed plasmid when the cell density reached approximately 80%influence. After culturing for another 48 h, the cells were washed with PBS, fixed with pre-cooled methyl alcohol for 10 min, and treated with 0.5% Triton X-100 for 20 min. Cells were blocked with goat serum for 30 min and incubated with primary antibody and Alexa Fluor®647-labeled IgG(H + L)/fluorescein isothiocyanates (FITCs) (Beyotime, Shanghai, China). The cells were counterstained with 4′,6-diamidino-2-phenylindole (DAPI, Beyotime, Shanghai, China). Fluorescent staining was performed and images were captured using a confocal microscope (Leica, Wetzlar, Germany).

### 2.12. Cell apoptosis

The fibroblasts were digested with trypsin and centrifuged at 3,000 rpm for 10 min. After adding pre-cooled PBS to the supernatant, the cells were centrifuged again at 1,000 rpm for 10 min. Then, binding buffer, Annexin V-FITC, and 7-Aminoactinomycin D were added to an Annexin V-FITC Apoptosis Detection Kit (BD Sciences, Franklin Lake, NJ, USA) in sequence and placed in the dark for 30 min at 4°C. Cell apoptosis was assessed within 1 h using a flow cytometer (BD FACSverse, BD Sciences, Franklin Lake, NJ, USA).

### 2.13. Statistical analysis

Statistical analysis was performed using SPSS 22.0 and GraphPad Prism (version 6.0; Boston, MA, USA), and Adobe Photoshop CS5 (San Jose, CA, USA) was used for plotting. Measurement data were expressed as the mean ± standard deviation, and comparisons between groups were performed using the independent samples *t*-test. *P* < 0.05 indicates a statistically significant difference.

## 3. Results

### 3.1. Establishment of rapid atrial pacing model

**Figure 1:**
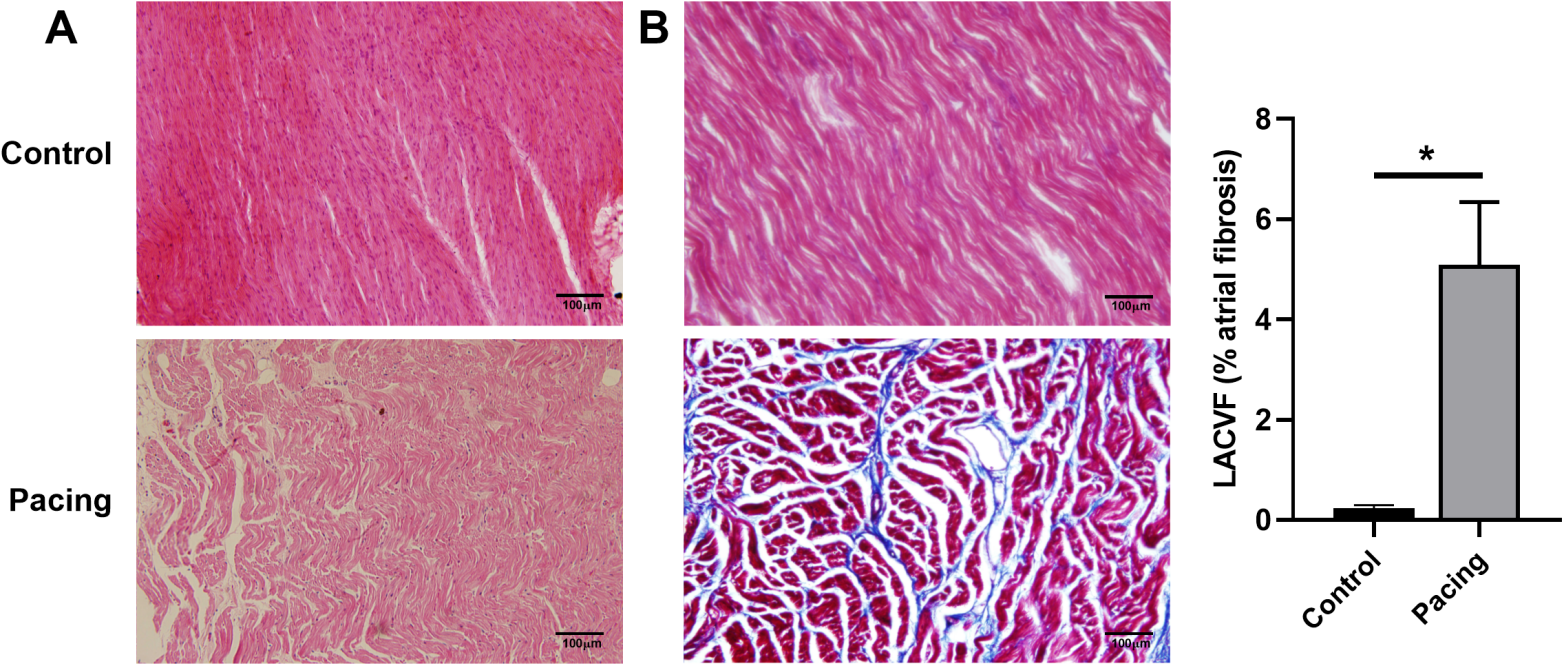
Establishment of a rapid atrial pacing model in dogs. (**A**) HE staining of the right atria from control and pacing group dogs. Clear myocardial fractures, disordered atrial myocytes, abnormal cell morphology, and increased intercellular space in the pacing group were observed. (**B**) Masson staining of the right atria from control and pacing group dogs. Collagen volume fraction was calculated. Data are expressed as the mean ± standard deviation, and comparisons between groups are performed using the independent samples *t*-test. *n* = 6, * indicates *P* < 0.05.

ECG showed that the sinus *P* waves disappeared and were replaced by fibrillation waves with varying amplitudes and frequencies (Supplementary Figure S1). In addition, HE staining revealed obvious myocardial fractures, disordered atrial myocytes, abnormal cell morphology, and increased intercellular spacing in the pacing group (Figure 1A). Masson staining indicated clear blue collagen deposition in the atrial myocyte space of the pacing group. The collagen volume fraction was significantly higher in the pacing group than that in the control group (*P* < 0.05; Figure 1B). These results indicate the successful establishment of a rapid atrial pacing model.

### 3.2. CircRNA expression profiles in the right atria of the rapid atrial pacing model

**Figure 2:**
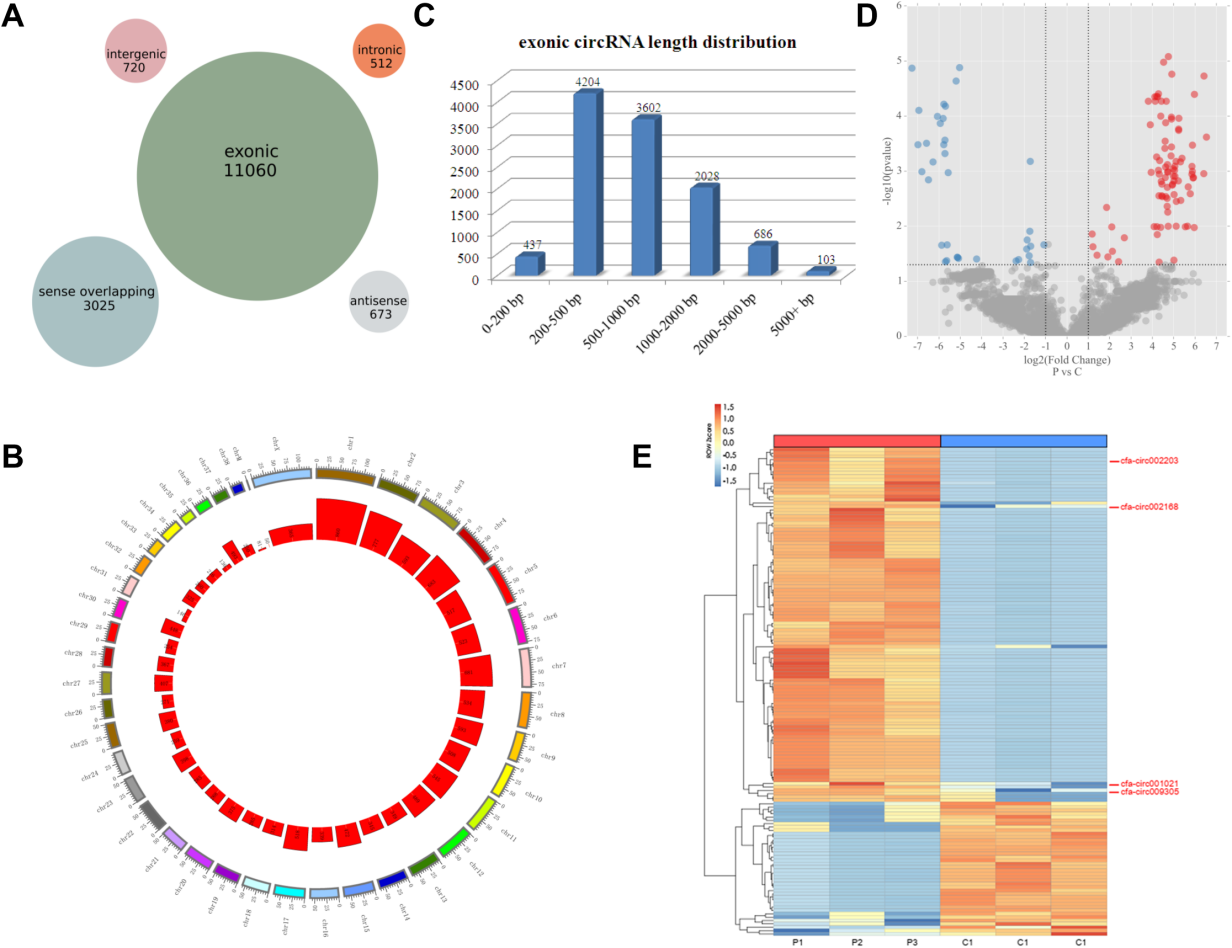
CircRNA expression profiles detected by high-throughput sequencing. (**A**) Number of circRNAs in the five classifications. (**B**) Distribution of circRNAs on the chromosome. (**C**) Number of exon circRNAs at each length interval. (**D**) Volcano plot of differentially expressed circRNAs (red plot represents upregulated circRNAs; blue plot represented downregulated circRNAs). (**E**) Heatmaps of the 146 differentially expressed circRNAs. The four circRNAs in the ceRNA network are indicated.

A total of 15,990 circRNAs were detected using high-throughput sequencing. According to the positions of the adjacent coding RNAs, the circRNAs obtained by sequencing could be roughly classified into five categories: exon circRNAs (11,060, 69%), justice overlap circRNAs (3,025, 19%), intergenic circRNAs (720, 5%), antisense circRNAs (673, 4%), and intron circRNAs (512, 3%) (Figure 2A). 15,990 circRNAs are widely distributed in all chromosomes including the mitochondrial genome (Figure 2B). The length of the circRNA exons ranged from 79 to 99,731 nucleotides, and that of most circRNAs (89%) ranged from 200 to 2,000 nucleotides. Only 1% of the circRNAs had lengths of over 5,000 nucleotides (Figure 2C). The analysis of different circRNA expression profiles showed 146 differentially expressed circRNAs in the pacing group compared with the control group, including 106 upregulated and 40 downregulated circRNAs (Figure 2D). Heatmap analysis showed that the 146 differentially expressed circRNAs could distinguish the samples in the control and pacing groups, suggesting the reliability of the differentially expressed circRNAs (Figure 2E).

### 3.3. Prediction of circRNA-miRNA-mRNA interactions

**Figure 3:**
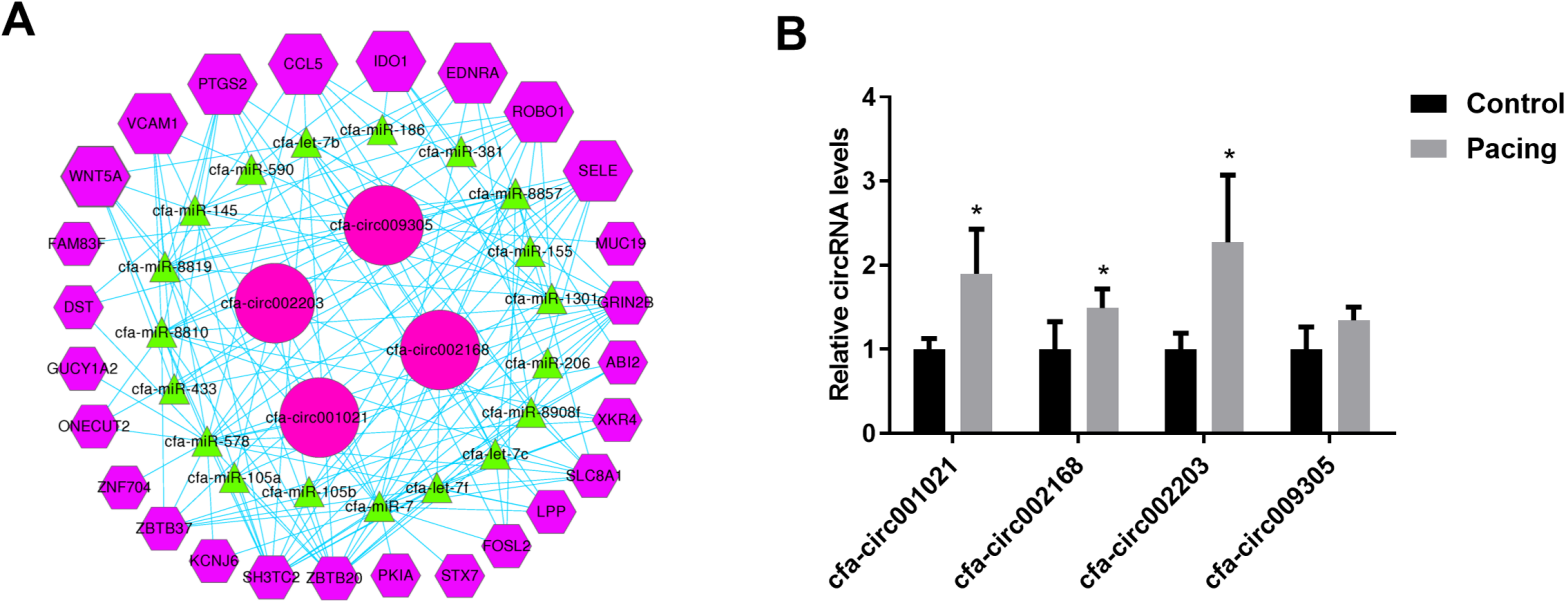
Prediction of circRNA-miRNA-mRNA interactions. (**A**) CeRNA network of circRNA-miRNA-mRNA. (**B**) Validation of four circRNAs in the ceRNA network by qRT-PCR. Compared with the control group, the expression levels of three circRNAs were significantly up-regulated in the pacing group. All experiments were conducted in triplicate. Data are expressed as the mean ± standard deviation, and comparisons between groups are performed using the independent samples *t*-test. *n* = 6, *P < 0.05.

We selected inflammation-related mRNAs from the literature (17–19) and constructed a ceRNA network between the top-25 differentially expressed circRNAs and inflammation-related mRNAs. Based on the abundance of binding miRNAs, free energy level, and size of the ceRNA score, several circRNAs with significant binding abilities were selected as representatives to draw the network diagram (Figure 3A), where a single circRNA interacted with different miRNAs in a family and different circRNAs also acted on the same miRNA (Figure 3A). qRT-PCR verification of the expression levels of the four circRNAs in the ceRNA network showed that, compared with the control group, the expression levels of three circRNAs were significantly upregulated in the pacing group (Figure 3B), including cfa-circ002203, cfa-circ002168, and cfa-circ001021. Cfa-circ002203, with the highest fold change, was selected to explore whether it was associated with fibrosis in atrial fibroblasts induced by Ang II *in vitro*.

### 3.4. Validation of cfa-circ002203 silenced and overexpressed cell line construction in Ang II-induced fibrosis

**Figure 4:**
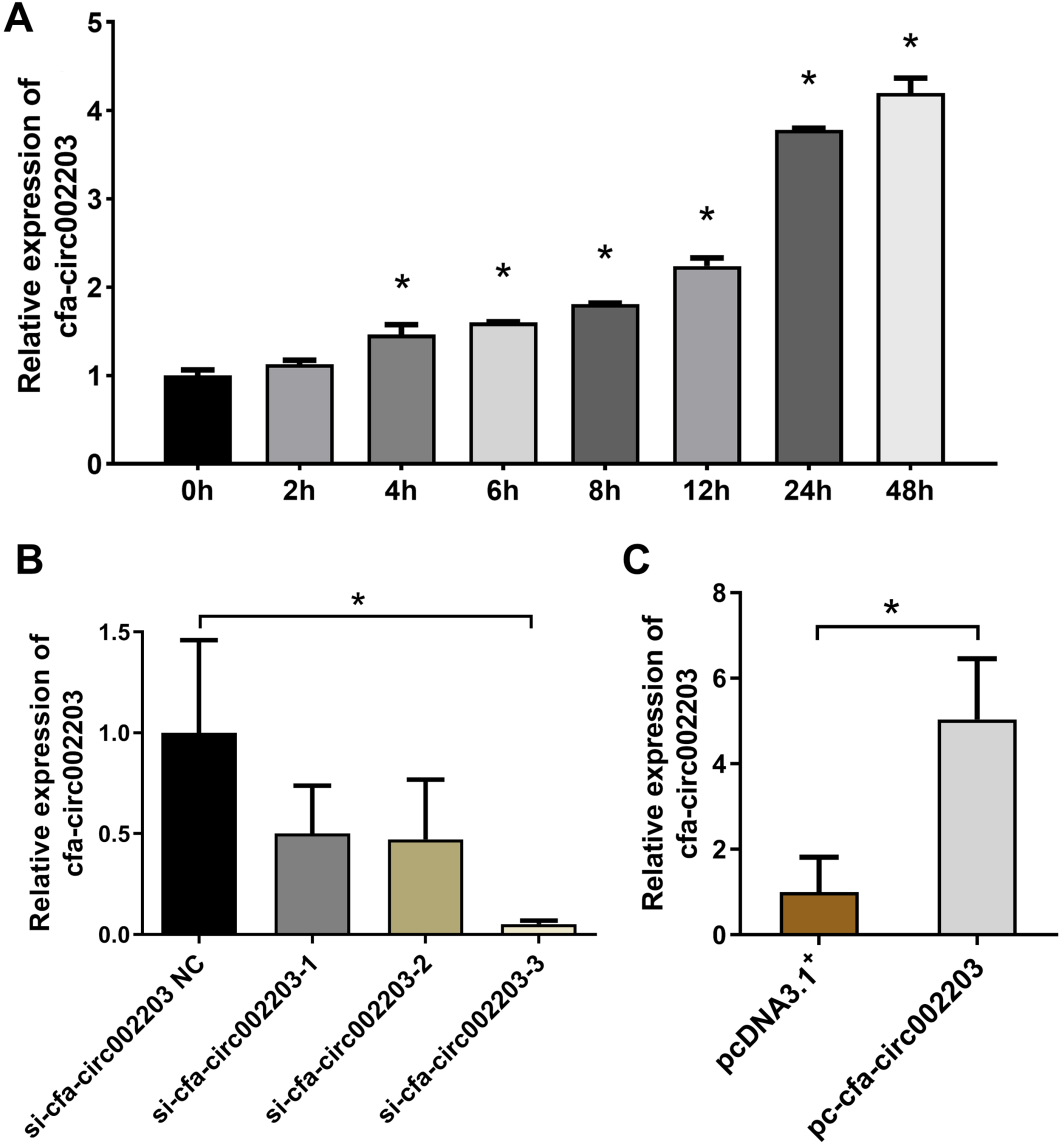
Cfa-circ002203 expression in atrial fibroblasts after cfa-circ002203 silencing or overexpression vector transfection. (**A**) Expression of cfa-circ002203 detected by qRT-PCR was significantly increased after AngII treatment with a time-dependent manner. (**B**) qRT-PCR showing that the cfa-circ002203 expression levels were significantly decreased after 48 h transfection of three siRNAs. (**C**) qRT-PCR showing that the cfa-circ002203 expression level was significantly increased after 48 h of pc-circRNA vector transfection. All experiments were conducted in triplicate. *P < 0.05.

Atrial fibroblasts may play a key role in myocardial fibrosis, and their proliferation leads to collagen remodeling. Therefore, we examined the effect of cfa-circ002203 on atrial fibroblasts. In Ang II-treated fibroblasts, the expression of cfa-circ002203 was significantly upregulated at time dependent manner (Figure 4A). Atrial fibroblasts with cfa-circ002203 silencing or overexpression were generated and verified by qRT-PCR. Among the three siRNAs of cfa-circ002203, si-cfa-circ002203-3 had the best silencing effect, with a 90% reduction (Figure 4B). The transfection of the cfa-circ002203-pcDNA3.1^+^ plasmid into atrial myofibroblasts significantly upregulated the cfa-circ002203 level (Figure 4C).

### 3.5. Cfa-circ002203 promoted the expression of inflammatory factors in atrial fibroblasts

**Figure 5:**
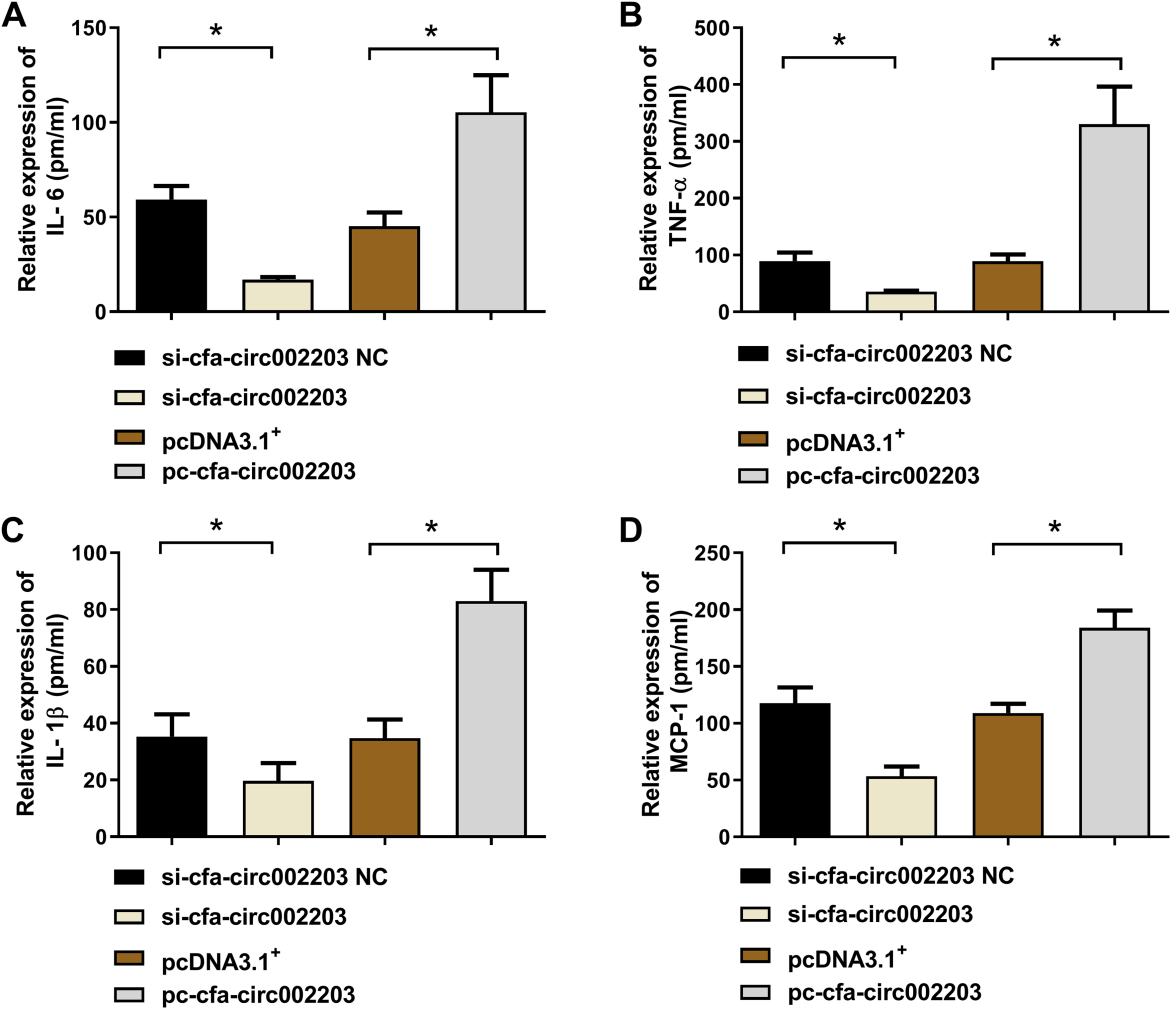
Expression levels of IL-6 (**A**), TNF-α (**B**), IL-1β (**C**), and MCP-1 (**D**) measured by ELISA after the silencing and overexpression of cfa-circ002203 in atrial fibroblasts. Compared with si-circRNA NC (negative control), the expression levels of IL-6, TNF-α, IL-1β, and MCP-1 were significantly decreased, while their levels were significantly increased after transfecting pc-circRNA compared with the empty vector, pcDNA3.1^+^. pm/ml, pmol/ml. *P < 0.05.

We collected supernatants of atrial fibroblasts transfected with si-cfa-circ002203 and overexpression vector, and then detected the changes in the proinflammatory factors interleukin (IL)-6, tumor necrosis factor (TNF)-α, monocyte chemoattractant protein-1 (MCP-1), and IL-1β. The results showed that the levels of IL-6, TNF-α, MCP-1, and IL-1β decreased significantly after transfection with si-cfa-circ002203 (Figures 5A–D), while they increased significantly after transfection with cfa-circ002203 overexpression vector (Figures 5A–D), indicating that cfa-circ002203 affected the secretion of inflammatory factors in myofibroblasts fibrosis induced by Ang II.

### 3.6. Cfa-circ002203 promoted fibroblast fibrosis and inhibited apoptosis

**Figure 6:**
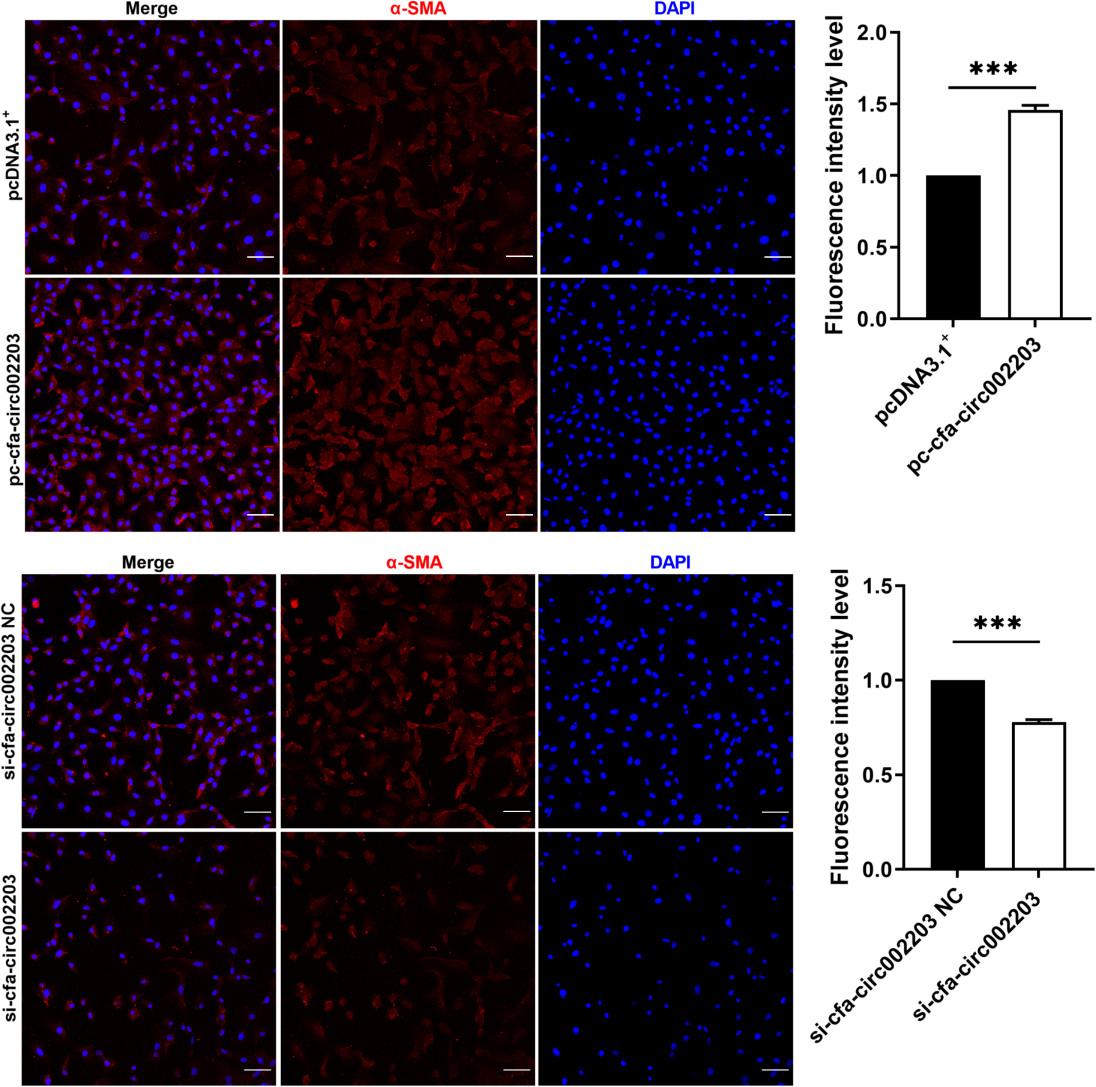
Immunofluorescence staining of α-SMA expression in atrial fibroblasts transfected with si-cfa-circ002203 or overexpression vector. α-SMA was labelled with Alexa Fluor®647-labeled IgG(H + L)/FITCs and cell nuclei were stained with DAPI. All experiments were conducted in triplicate. Scale bar, 20 µm. Data are expressed as the mean ± standard deviation, and comparisons between groups are performed using the independent samples *t*-test. ***P < 0.001.

In fibroblast fibrosis induced by Ang II, we detected cells using a confocal laser microscope and found that the expression of α-SMA decreased significantly after the cells were transfected with si-cfa-circ002203, while it was significantly upregulated after transfection with the cfa-circ002203 overexpression vector (Figure 6).

**Figure 7:**
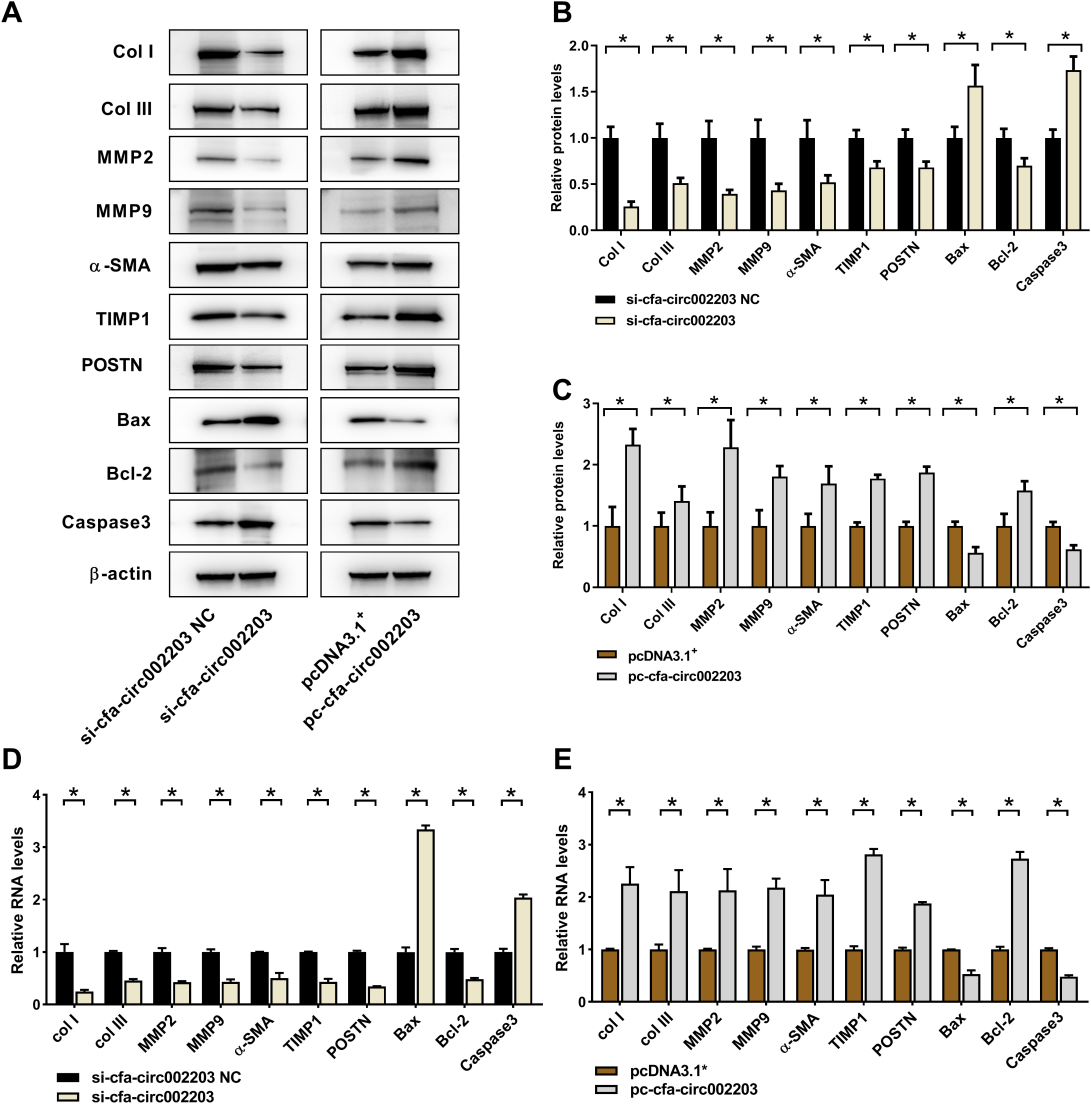
Protein and mRNA expression levels of fibrosis- and apoptosis-associated proteins in atrial fibroblasts transfected with cfa-circ002203 silencing or overexpression vectors. (**A**) Representative western blotting images. (**B,C**) Relative expression of proteins calculated from three independent samples in cfa-circ002203-silencing and cfa-circ002203-overexpressing cells. (**D,E**) Relative expression of fibrosis- and apoptosis-associated genes detected by qRT-PCR. Data are expressed as the mean ± standard deviation, and comparisons between groups are performed using the independent samples *t*-test. *n* = 3, *P < 0.05.

Western blotting and qRT-PCR were used to detect the expression of the fibrosis indicators collagen I, collagen III, MMP2, MMP9, α-SMA, TIMP1 and POSTN as well as the apoptosis factors Bax, Bcl-2, and Caspase 3. The results showed that the expression of fibrosis indicators was significantly decreased after transfection with si-cfa-circ002203 compared with that in the control (Figures 7A,B,D) and was significantly upregulated after transfection with the si-cfa-circ002203 overexpression vector (Figures 7A,C,E). These results suggest that cfa-circ002203 might be important in promoting the differentiation of fibroblasts into myofibroblasts. In addition, the expression of Bax and Caspase3 was significantly upregulated after transfection with si-cfa-circ002203 compared with that in the control group (Figures 7A,B,D), while it was significantly decreased when cfa-circ002203 was overexpressed (Figures 7A,C,E). The expression of Bcl-2 showed the opposite trend. These results suggest that cfa-circ002203 represses fibroblast apoptosis.

### 3.7. Effects of cfa-circ002203 on the proliferation and migration of fibroblasts

**Figure 8:**
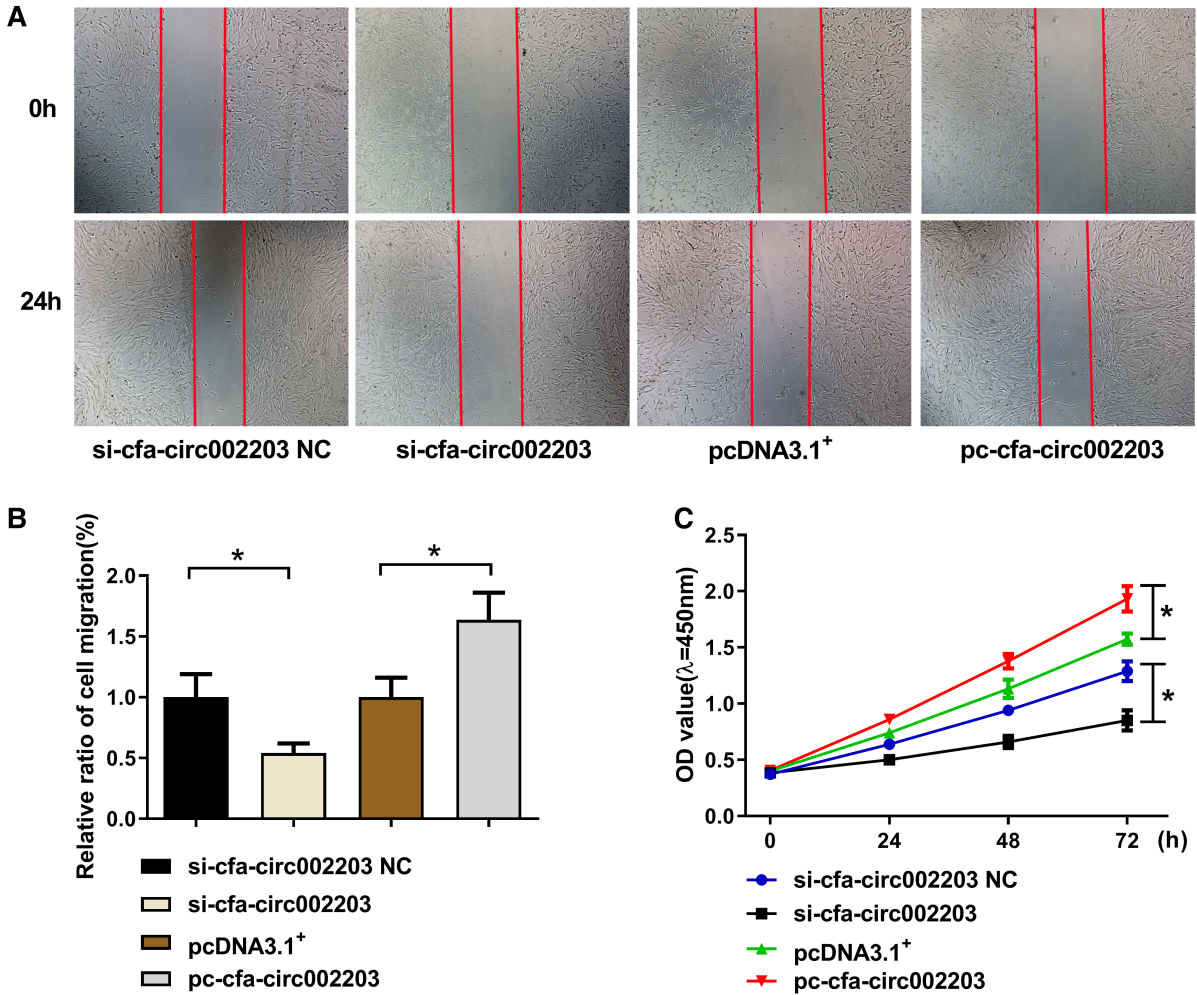
Effects of cfa-circ002203 on the proliferation and migration of fibroblasts. (**A,B**) The cell scratch assay was used to detect the migration of fibroblasts after silencing and overexpressing cfa-circ002203. The red lines depict the edges of cells. The mobility of the si-cfa-circ002203 group was lower than that of the si-cfa-circ002203 NC group and increased after the overexpression of cfa-circ002203. (**C**) CCK8 was used to detect the proliferation of fibroblasts. All experiments were conducted in triplicate. *P < 0.05. Compared with the NC group, the proliferation ability of fibroblasts was weakened after transfecting with si-cfa-circ002203, and the proliferation ability of cfa-circ002203-overexpressing fibroblasts was significantly increased.

A cell scratch assay was performed to detect fibroblast migration after the silencing and overexpression of cfa-circ002203, and the CCK8 assay was used to detect fibroblast proliferation. The results showed that, 24 h after the cell scratching experiment, the mobility of the si-cfa-circ002203 group was lower than that of the si-cfa-circ002203 NC (negative control) group and increased after the overexpression of cfa-circ002203 (Figures 8A,B). The CCK8 results showed that, compared with the NC group, the proliferative ability of fibroblasts was weakened after transfection with si-cfa-circ002203, and the proliferative ability of cfa-circ002203-overexpressed fibroblasts was significantly increased (Figure 8C).

### 3.8. Effects of cfa-circ002203 on fibroblast apoptosis

**Figure 9:**
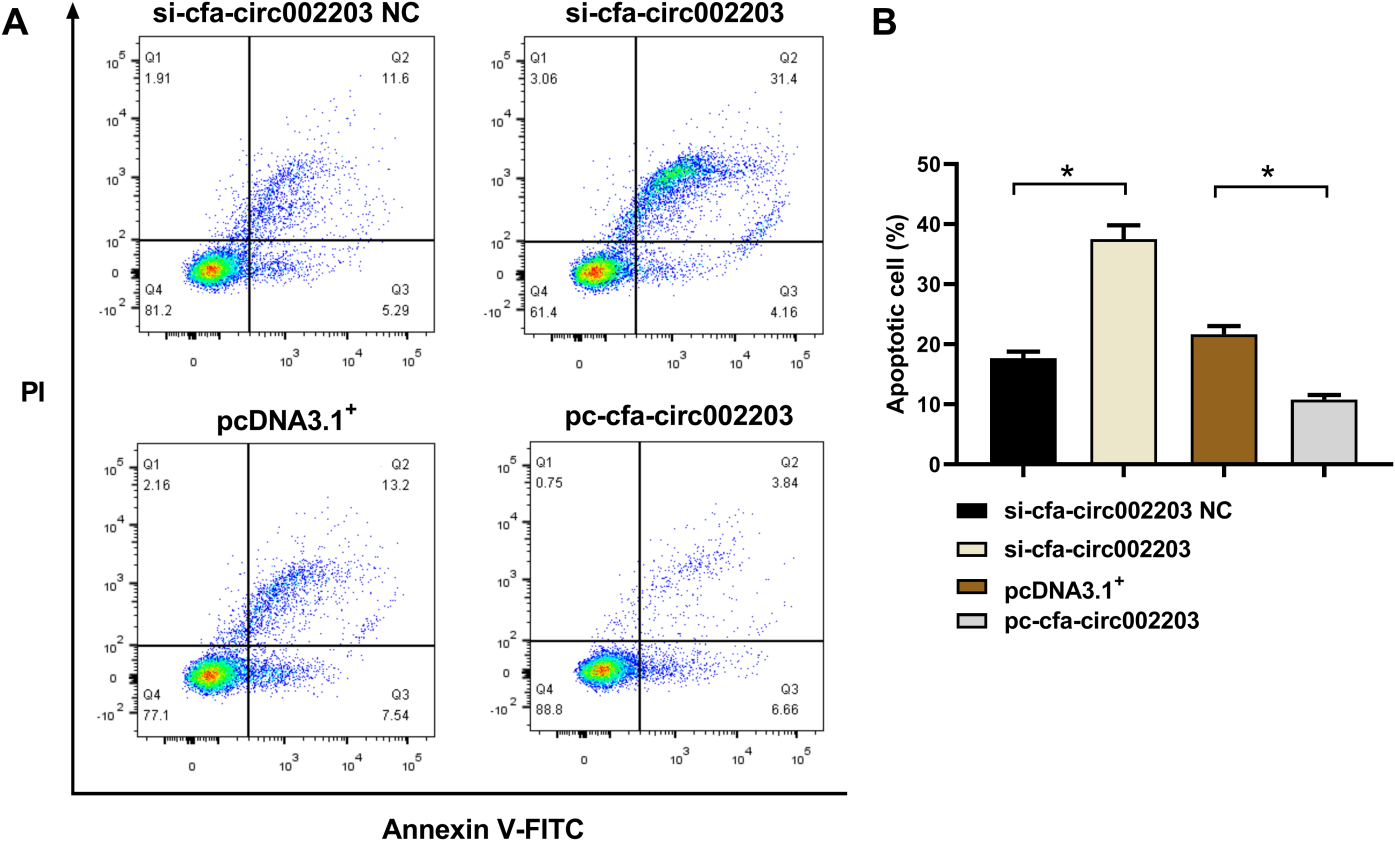
Effects of cfa-circ002203 on fibroblast apoptosis detected by flow cytometry. (**A**) Representative picture of fibroblast apoptosis detected by flow cytometry. (**B**) Quantitative analysis of apoptotic cells in each group. All experiments were conducted in triplicate. Compared with the control group, si-cfa-circ002203 increased the number of late apoptotic cells in Q2 and decreased the number of living cells in Q1. In contrast, the overexpression of cfa-circ002203 inhibited fibroblast apoptosis. *P < 0.05.

Fibroblasts were stained with Annexin V/PI, and cell apoptosis was measured by flow cytometry. Compared with the control group, si-cfa-circ002203 increased the number of late apoptotic cells in Q2 and decreased the number of living cells in Q1 (*P* < 0.05). In contrast, the overexpression of cfa-circ002203 inhibited fibroblast apoptosis (*P* < 0.05; Figure 9).

## 4. Discussion

The regulatory and functional roles of circRNAs in the progression of heart disease have been proposed (8, 20) and their regulatory role in AF-induced fibrosis is the focus of the present study. This is the first study to demonstrate that cfa-circ002203 plays an important role in AF-induced atrial fibrosis and is critical for the progression of atrial fibroblast inflammation and fibrosis.

Fibrosis is part of the pathological remodeling of atrial tissue and clinical cardiac disease. Although corresponding treatments have been adopted according to the different mechanisms of AF, there is currently no effective treatment for atrial fibrosis, and the prevention and reversal of atrial fibrosis is still a major problem in medicine (6). CircRNA is a potential target for preventing or even reversing the progression of tissue fibrosis, because previous studies have reported the role of circRNAs in inhibiting myocardial fibrosis. For instance, circRNA_000203 regulates the expression of fibrosis-associated genes in cardiac fibroblasts (21). circHIPK3 and mmu_circ_0005019 prevent the proliferation and migration of cardiac fibroblasts (11, 22). These data reveal the function of circRNAs in influencing the expression of fibroblast-related genes and the phenotypic change from fibroblasts to fibrotic phenotypes. In our experiments using high-throughput sequencing of fibrotic atrial tissue in an AF dog model, we found that 15,990 circRNAs were widely distributed in all chromosomes, and 146 circRNAs were dysregulated in AF-induced fibrosis, indicating the potential of circRNAs to participate in AF and AF-induced myocardial fibrosis.

Although circRNAs have been suggested to perform various biological functions, such as acting as miRNAs or protein inhibitors (“sponges”), regulating protein functions, or self-translation (23), their regulatory networks with miRNAs and mRNAs are widely accepted to play key roles in disease progression, including cancer and immune system, metabolic, and endocrine diseases (24–27). This also applies to the molecular mechanisms underlying cardiovascular diseases (28, 29). Based on the circRNA-miRNA-mRNA network, we screened circRNAs that might have affected the expression of atrial fibrosis-related mRNA and verified them in the atrial tissues of the AF dog model. Finally, cfa-circ002203 was selected as a candidate gene for the further analysis of the involvement of circRNAs in fibroblast fibrosis. Cfa-circ002203 is located on chr20:33297388–33298499+ in the dog genome, and its function has not yet been elucidated. Therefore, we investigated the role of cfa-circ002203 in Ang II-induced fibrosis.

First, cfa-circ002203 is thought to be associated with fibroblast inflammation, which is an important factor in the occurrence, maintenance, and recurrence of AF (30). In atrial tissue from patients with AF, there was a significant, positive correlation between the serum proinflammatory cytokine TNF-α and IL-6 levels and the collagen volume fraction (31). Fibrosis is a pathological process of AF substrate formation and is affected by inflammation (32). Our data showed that the Ang-II-induced expressions of proinflammatory cytokines IL-6, TNF-α, IL-1β, and chemokine MCP-1 in fibroblasts changed, along with cfa-circ002203 silencing and overexpression. This led to the interesting speculation that cfa-circ002203 is an active regulator of the inflammatory activation of fibroblasts.

Atrial fibrosis entailing fibrous collagen types I and III is a typical characteristic of AF. Collagen is a relatively hard material with high tensile strength, and even small changes in its quality, indicated by its concentration, proportion, and degree of crosslinking, have been proven to significantly affect cardiac functional properties, resulting in diastolic and contractile properties (33). Our results showed that the expression of collagen types I and III significantly decreased in the cfa-circ002203-silenced fibroblasts, and were up-regulated in cfa-circ002203-overexpressed fibroblasts. Furthermore, we examined the expression of MMP-2 and MMP-9, proteins in fibrotic fibroblasts that are essential for normal and pathological tissue remodeling, by controlling the degree of ECM remodeling (34). Our data showed that the silencing or overexpression of cfa-circ002203 promoted the downregulation and upregulation of MMP-2 and MMP-9 expression, respectively. It Cfa-circ002203 appears to function in the ECM remodeling of fibroblasts.

Additionally, we verified the role of cfa-circ002203 in the transformation of fibroblasts into activated myofibroblasts. The transformation from fibroblasts to myofibroblasts occurs during the late proliferative stage and is characterized by the expression of SMA (35). Our data suggest that α-SMA expression and cell migration induced by Ang II changed with the silencing or overexpression of cfa-circ002203. Collectively, cfa-circ002203 exerts an active function on the expression levels of the remarkable proteins of fibrotic fibroblasts, including collagen I, collagen III, MMP2, MMP9, and α-SMA, and is considered as a promotor of the fibrosis of fibroblasts.

We also examined the effect of cfa-circ002203 on apoptosis. Western blotting showed that the expression of Bax and Caspase3 was significantly upregulated after transfection with si-cfa-circ002203, whereas it was significantly decreased when cfa-circ002203 was overexpressed compared with the control group. The expression of Bcl-2 showed the opposite trend. In addition, flow cytometry showed that the overexpression of cfa-circ002203 decreased the number of late apoptotic cells in Q2 and increased the number of living cells in Q1 compared with the control group (*P* < 0.05). AF may result in cardiac apoptosis by downregulating protective mechanisms and activating proapoptotic pathways. Xu et al. identified increased levels of BAX and lower levels of BCL-2 in AF and determined that the protein expression of BAX and BCL-2 was correlated with the frequency of apoptosis in AF (36). These results suggest that cfa-circ002203 promotes the apoptosis of fibroblasts and may serve as a target for the treatment of AF.

This study had some limitations. First, the role of cfa-circ002203 on cell proliferation was not checked by EDU incorporation assay. Second, the expression of circRNA_002203 over the timecourse of the proliferation assay should also be checked. Therefore, further research is required to investigate the role of circ002203 in fibrosis.

In summary, the results of the present study suggest that several circRNAs exhibit aberrant expression patterns in canine AF-induced fibrosis. Among these circRNAs, cfa-circ002203 was closely associated with fibrosis, inflammatory activation, and fibroblast apoptosis. The findings of this study strengthen the role of circRNAs in AF-induced fibrosis in dogs and provide a perspective on the disease mechanisms and therapeutic targets for AF.

## Data Availability

The original contributions presented in the study are publicly available. This data can be found here: https://www.ncbi.nlm.nih.gov/geo/, GSE225086.

## Supplementary material

SUPPLEMENTARY FIGURE S1

ECG of the body surface (III lead) and epicardium (LAA, left atrial appendage; RAA, right atrial appendage; LLA, low left atrial; LRA, low right atria; HLA, high left atria; HRA, high right atria).
