# Supplementary figures and images for: Cfa-circ002203 was upregulated in rapidly paced atria of dogs and involved in the mechanisms of atrial fibrosis
Source: Front Cardiovasc Med. 2023 Aug 1;10:1110707. doi: 10.3389/fcvm.2023.1110707 (PMC10427503; doi:10.3389/fcvm.2023.1110707)

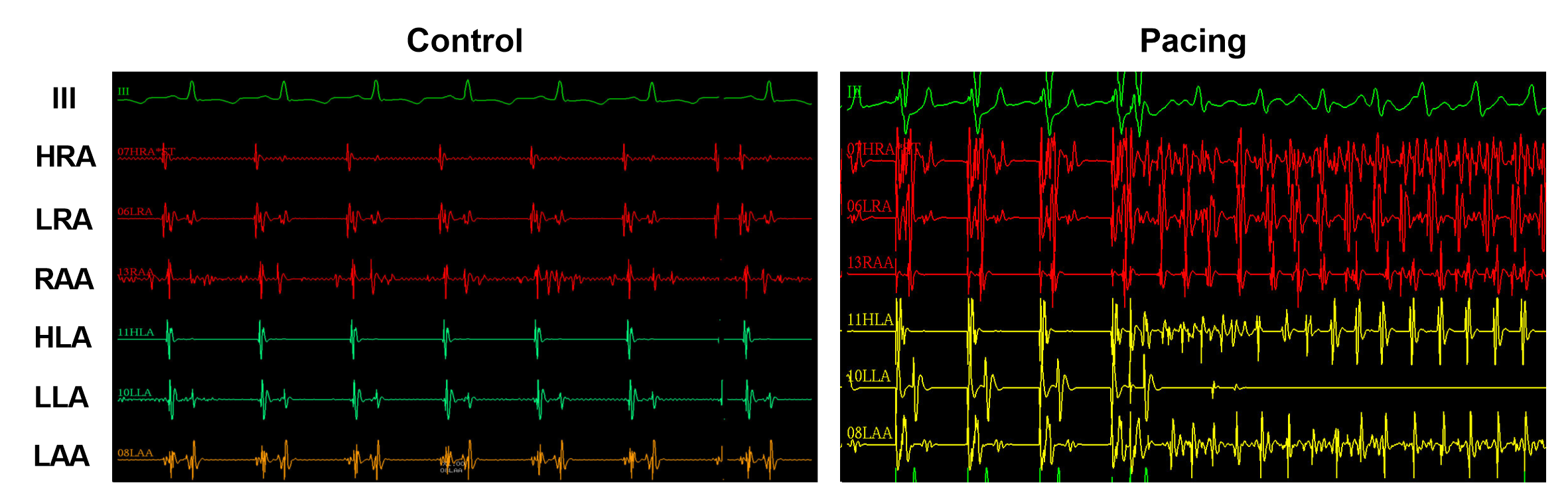

Supplement: Supplementary file 1 [file Image1.tif]
